# Ephs in cancer progression: complexity and context-dependent nature in signaling, angiogenesis and immunity

**DOI:** 10.1186/s12964-024-01580-3

**Published:** 2024-05-29

**Authors:** Xiaoting Guo, Yanyi Yang, Jingqun Tang, Juanjuan Xiang

**Affiliations:** 1 Hunan Key Laboratory of Early Diagnosis and Precise Treatment of Lung Cancer, the Second Xiangya Hospital, Central South University, Changsha, 410013 Hunan China; 2 Department of Thoracic Surgery, the Second Xiangya Hospital, Central South University, Changsha, 410013 Hunan China; 3 Cancer Research Institute, School of Basic Medical Science, Central South University, Changsha, Hunan China; 4 NHC Key Laboratory of Carcinogenesis and the Key Laboratory of Carcinogenesis and Cancer Invasion of the Chinese Ministry of Education, Xiangya Hospital, Central South University, Changsha, Hunan China; 5 Health Management Center, the Second Xiangya Hospital, Central South University, Changsha, Hunan China

**Keywords:** Eph/ephrin, Vascular, Immune, Tumor, Pain

## Abstract

Eph receptors constitute the largest family of receptor tyrosine kinases, comprising 14 distinct members classified into two subgroups: EphAs and EphBs.. Despite their essential functions in normal physiological processes, accumulating evidence suggests that the involvement of the Eph family in cancer is characterized by a dual and often contradictory nature. Research indicates that Eph/ephrin bidirectional signaling influences cell–cell communication, subsequently regulating cell migration, adhesion, differentiation and proliferation. The contradictory functionalities may arise from the diversity of Eph signaling pathways and the heterogeneity of different cancer microenvironment. In this review, we aim to discuss the dual role of the Eph receptors in tumor development, attempting to elucidate the paradoxical functionality through an exploration of Eph receptor signaling pathways, angiogenesis, immune responses, and more. Our objective is to provide a comprehensive understanding of the molecular mechanisms underlying tumor development. Additionally, we will explore the evolving landscape of utilizing Eph receptors as potential targets for tumor therapy and diagnostic tools.

## Introduction

The human erythropoietin-producing hepatocellular (Eph) receptors consitute the largest family of receptor tyrosine kinases (RTKs), comprising 14 distinct members classified into two subgroups: EphAs and EphBs. The EphA subgroup includes nine receptors (EphA1-8 and EphA10), while the EphB subgroup consists five members (EphB1-4 and EphB6) [1]. Each member is comprised of an extracellular domain, a transmembrane region, and an intracellular tyrosine kinase domain. Eph receptors initiate signal transduction by interacting with their ligands, ephrins. These receptors are located on cell surfaces and transmit signals upon binding to the ligands [2]. EphA receptors predominantly bind to glycosylphosphatidylinositol (GPI) -linked ephrin-A ligands, whereas EphB receptors bind to ephrin-B ligands possessing transmembrane and intracellular domains [3]. Additionally, EphA4 and EphB2 receptors can also bind eprhins of a different class [4].

Eph/ephrin signaling displays at least two unique characteristics compared to other RTKs. Firstly, it exhibits bidirectional signaling, transmitting signals within both the receptor- and ligand-expressing cells upon binding to the membrane-bound ligand (forward and reverse signalings) [1]. Secondly, Eph receptors may function in a kinase activity-independent mechanism. The Eph/ephrin system is influenced not only by their own interactions but also by other RTKs [5].

Throughout various developmental processes, Eph/ephrin signaling plays diverse biological roles, including growth cone retraction in axon guidance, the formation of synaptic connections between neurons, cell sorting in embryo patterning, cell migration, platelet aggregation, and blood vessel remodeling [4, 6]. They regulate essential processes such as cell adhesion, migration, and cell growth. Despite its crucial functions in normal physiological processes, mounting evidence suggests that the Eph family's involvement in cancer is characterized by a dual and often contradictory role [7]. On one hand, some studies propose that the Eph family exhibits tumor-suppressive effects in certain tumor types, inhibiting tumor cell proliferation, invasion, and metastasis, thereby impeding tumor development. Conversely, an increasing body of evidence indicates that Eph receptors are associated with adverse prognosis, facilitating tumor growth and metastasis in other tumor types. Recent studies have focused on the functions of Eph/ephrin signaling in vasculogenesis and immune modulation [8]. However, the mechanism of Eph/ephrin involvement in cancer metastasis, invasion and angiogenesis remains to be fully understood [9]. The contradictory functionality may arise from the diversity of Eph signaling pathways and the heterogeneity of the different cancer microenvironments. In this review, our objective is to elucidate the dual role of Eph receptors in tumor development, attempting to explain the paradoxical functionality through an exploration of Eph receptor signaling pathways, angiogenesis, immune responses, and more. We aim to provide a deeper understanding of the molecular mechanisms underlying tumor development. Additionally, we will explore the evolving landscape of using Eph receptors as targets for tumor therapy and diagnostic tools.

## The dual role of Eph family in cancer development

Tumorigenesis is a complex process characterized by distinct hallmarks extensively studied and expanded to encompass 10 organizing principles, including sustaining proliferative signaling, evading growth suppressors, resisting cell death, enabling replicative immortality, inducing angiogenesis, activating invasion and metastasis, genome instability, inflammation, reprogramming of energy metabolism, and evading immune destruction [10]. The tumor microenvironment also plays a critical role in increasing the complexity of tumorigenesis [10].

**Fig. 1:**
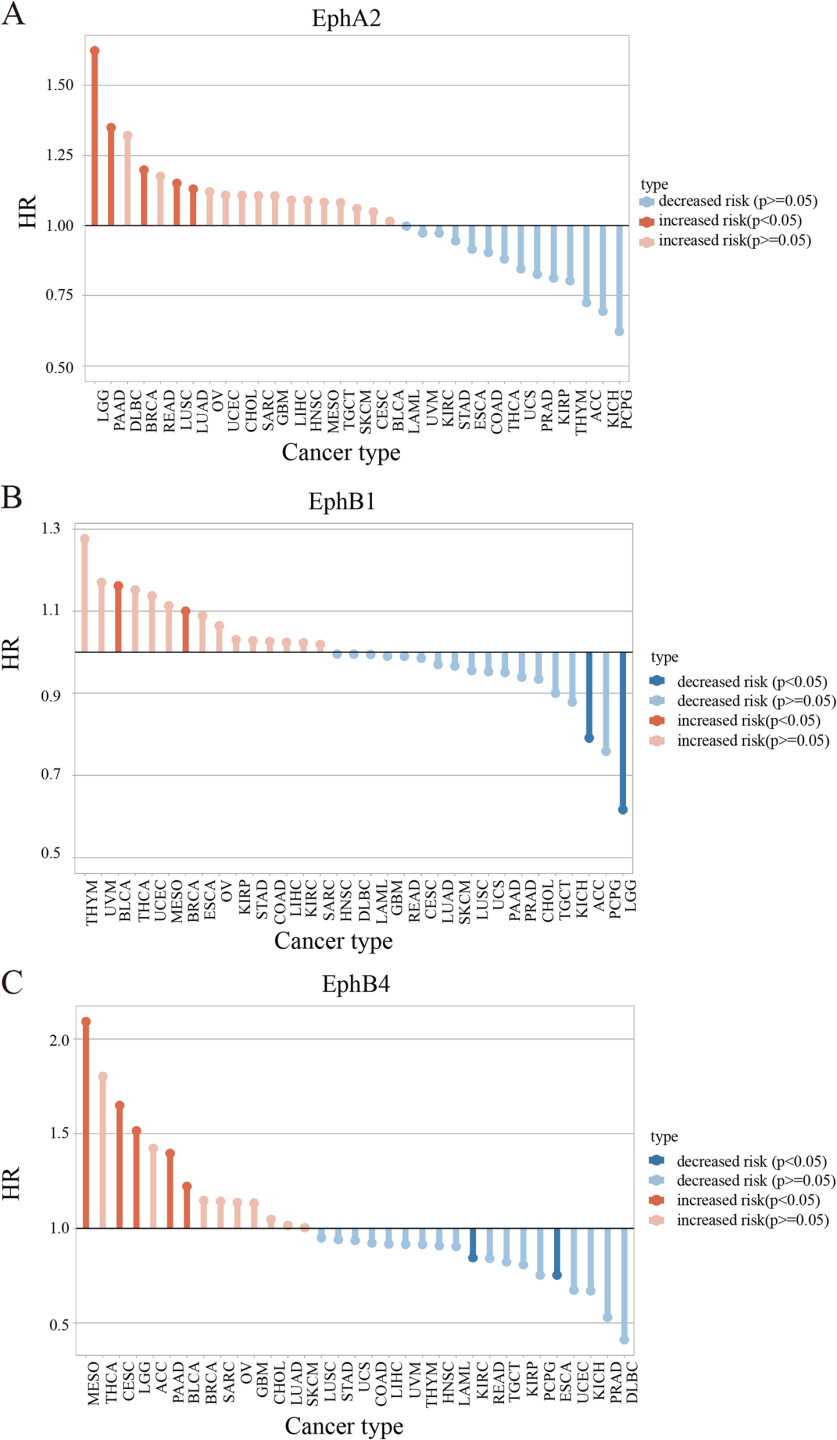
The Hazard Ratio of survival events associated with Eph receptor expressions across different cancer types. The TISCH2 database (http://tisch.compgenomics.org/), containing scRNA-seq data from more than 50 cancer types, was utilized to analyze the association of Eph receptor expression and HR of survival. An HR > 1 indicates a high risk of death, while an HR < 1 indicates a low risk of death. **A** HR of patient survival according to the expression of EphA2 in distinct cancer types from datasets. **B** HR of patient survival according to the expression of EphB1 in distinct cancer types from datasets. **C** HR of patient survival according to the expression of EphB4 in distinct cancer types from datasets

The Eph family of receptor tyrosine kinases has emerged as a crucial player in tumorigenesis, displaying a diverse range of roles in tumor development and progression. The function of Eph receptors and their ligands extends beyond a binary classification of either tumor suppression or tumor promotion (Table 1). Instead, they play diverse roles in different types of tumors, underscoring their complexity and context-dependent nature [8]. As depicted in Fig. 1, the Eph receptor expression exhibits a correlation with cancer patient survival. The Hazard Ratio (HR) indicates the variability of Eph functions across different cancer types (Fig. 1). To ensure that survival analyses involving multiple tumor groups were presented, the figures also displayed those with *P*-values exceeding 0.05.

**Table 1:** The Dual role of Eph family in tumorigenesis

Cancer Type	Eph/Ephrin Type	Function in Tumorigenesis	Role of Eph/Ephrin	References
Breast cancer	EphA2	Tumor-promoting	The malignant behavior of EPHA2 is mediated by trans signaling	[11]
	EphA2	Tumor-suppressive	Its antioncogenic properties are attributed to cis signaling, which required phosphorylation of EphA2 on serine 897 by Akt	[11]
	EphA4	Tumor-promoting	EphA4-mediated juxtacrine signaling maintains the stem cell state through their interactions with tumor-associated monocytes/macrophages and CSCs	[12]
	EphB4	Tumor-suppressive	The Abl–Crk pathway inhibits breast cancer cell viability and proliferation in addition to motility and invasion, and also downregulates the pro-invasive matrix metalloprotease, MMP-2	[13]
	Ephrin-A1	Tumor-promoting	EphA2-Ephrin A1 reverse signaling	[14]
	Ephrin-B2	Tumor-promoting	May participate in positioning and pattern formation in the adult mammary gland	[15]
Liver cancer	EphA1	Tumor-promoting	Promotes angiogenesis in tumor tissue, and is related to VEGF	[16]
	EphA2	Tumor-promoting	EphA2 is related to VEGF signaling and inhibits many angiogenic activities of VEGF	[17]
	EphA5	Tumor-promoting	EphA5 are essential to sustain the survival of HCC cells through downstream AKT‐dependent, ERK‐dependent, and p38‐dependent signaling cascades	[18]
	EphrinA2	Tumor-promoting	EphrinA2 endowed cancer cells with resistance to tumor necrosis factor alpha-induced apoptosis, thus facilitating their survival	[19]
	EphrinA3	Tumor-promoting	Ephrin-A3 directly binds with EphA2 and induces autophosphorylation of tyrosine residues (Tyr588)	[20]
	EphrinA4	Tumor-promoting	EFNA4 influenced the proliferation and migration of HCC cells through a PIK3R2/GSK3β/β-catenin positive feedback loop	[21]
	EphrinB1	Tumor-promoting	The expression of ephrin-B1 promotes tumor growth by initiating tumor angiogenesis in HCC	[22]
	EphrinB2	Tumor-promoting	Ephrin-B2 and Dll4 are involved in neoangiogenesis in HCC	[23]
Prostate cancer	EphA2	Tumor-promoting	EphA2 is related to VEGF signaling and inhibits many angiogenic activities of VEGF	[17]
	EphA5	Tumor-promoting	The hypermethylation of EphA5 was significantly associated with the downregulation of EphA5	[24]
	EphA6	Tumor-promoting	EphA6 may promote prostate cancer progression and metastasis by enhancing angiogenesis	[25]
	EphB4	Tumor-promoting	EphB4 and integrin β8 may work together to control cell movement	[26]
	EphrinB1	Tumor-promoting	Expression of ephrin-B1 was significantly induced by slug via the E-box motif and promoted cell migration and invasion	[27]
Esophageal squamous cell carcinoma	EphA2	Tumor-promoting	EphA2 is related to VEGF signaling and inhibits many angiogenic activities of VEGF	[17]
	EphB3	Tumor-promoting	EphB3 inhibition can regulate EMT through the AKT pathway to limit tumor migration and invasion of ESCC cancer	[28]
	EphB4	Tumor-promoting	The EphB4 forward signaling can promote the proliferation and migration of endothelial cells via the Pl-3K pathway	[29]
	EphrinA1	Tumor-promoting	EFNA1 induction requires activation of JNK and p38MAPK signaling pathways, which can regulate actin recombination and cell migration in endothelial cells	[30]
Ovarian cancer	EphA2	Tumor-promoting	EphA2 is related to VEGF signaling and inhibits many angiogenic activities of VEGF	[17]
Melanoma	EphA2	Tumor-promoting	EphA2 is related to VEGF signaling and inhibits many angiogenic activities of VEGF	[17]
	EphrinA1	Tumor-promoting	The direct action of inflammatory cytokines produced in advanced lesions and genetic selection of tumor cells that produce this factor, known to be both angiogenic and a growth factor for melanoma cells	[31]
Gastric cancer	EphA1	Tumor-promoting	Promotes inflammation and angiogenesis and is associated with IL-6 and VEGF	[32]
	EphA2	Tumor-promoting	Regulates Wnt/β-catenin signaling and promote cell proliferation	[33]
	EphA4	Tumor-promoting	EphA4 has been shown to phosphorylate FGFR2 and 3, and FGFR-2 and FGFR-4 mRNA overexpression in gastric cancer	[34]
	EphrinA1	Tumor-promoting	Phosphorylation of EPHA1 further enhanced cell migration mediated by soluble Ephrin A2	[35]
	EphrinB1	Tumor-promoting	Tyrosine phosphorylation of ephrin-B1 can inhibite tumor cell invasion, as signaling mediated by ephrin-B1 promoted the intracellular transport and secretion of matrix metalloproteinases	[36]
Renal cell carcinomas	EphA4	Tumor-promoting	EphA4 induces adhesion junction disturbances, including e-cadherin internalization and downregulation	[37]
Non-Small Cell Lung Carcinoma	EphA2	Tumor-promoting	The EphA2 invasion signal is attributed to the G391R mutation and subsequent phosphorylation of two serine residues within mTOR	[38]
	EphA4	Tumor-suppressive	Affects cancer cell migration and invasion	[39]
	EphA5	Tumor-suppressive	Associated with the ability of tumors to proliferate	[39]
	EphA7	Tumor-suppressive	Associated with the ability of tumors to proliferate	[39]
	EphB1	Tumor-promoting	Ligand-independent EphB1 promotes epithelial-mesenchymal transition (EMT) by upregulating CDH2	[40, 41]
	EphB4	Tumor-promoting	EphB4 increases cell viability through the phosphatidylinositol 3-kinase /Akt signaling pathway	[42]
	Ephrin-B3	Tumor-promoting	Both Akt Ser 129 and p38MAPK indicate the potential to drive migration/proliferation	[43]

### Promoting tumor progression

Numerous studies have reported the overexpression of Eph receptors and ephrins in various types of cancer, including breast cancer, gastrointestinal cancer, melanoma and small-cell lung carcinoma (SCLC) [44]. These molecules exhibit increased levels in tumors, especially during the more aggressive stages of tumor progression [45]. For instance, EphA2, expressed at minimal levels in normal human breast epithelium [46], is significantly overexpressed in 60% to 80% of breast cancers [47]. Additionally, EphA2 upregulation has been observed in liver, prostate cancer, glioblastoma, esophageal squamous cell carcinoma, ovarian cancer, and melanoma, with the highest levels detected in the most invasive tumor cells [17]. Conversely, inhibiting EphA2 expression using small interfering RNA (siRNA) has been shown to impede the malignant progression of pancreatic, ovarian, and mesothelioma tumor cell lines, underscoring the pivotal role of EphA2 in tumor development [48]. In small cell lung cancer (SCLC) cell lines and tumor samples, transcripts encoding members of EphB receptors and ephrins are coexpressed. In human leukemia-lymphoma cell lines, both the EphB4 receptor and ephrinB2 ligand are commonly co-expressed, with EphB4 mRNA detected in 68/70 cell lines and 58/70 cell lines displaying positive ephrinB2 mRNA expression [49]. Although evidence suggests that the interaction between Eph receptors and other oncogenic pathways promotes tumor cell malignancy, this might occur in an ephrin-independent manner [5, 50, 51]. Of note, a paradoxical early response in the activation of cell surface receptors is the downregulation of these receptors, which involves ligand-stimulated endocytosis. However, the activity of RTK within intracellular lysosome following their internalization remains to be discussed [52].

### Inhibiting tumor development

Numerous studies have provided compelling evidence supporting the role of Eph receptors in tumor suppression [50]. Specifically, EphB1 has been found to be down-regulated in gastric cancer, renal cell carcinomas and colorectal cancer [53, 54]. Additionally, there is a strong correlation between the loss of EphB1 protein and both metastasis and reduced survival rates in patients with serous ovarian cancer [55]. Ligand-dependent EphB1 signaling has been shown to effectively suppress glioma invasion and is positively correlated with patient survival [56]. In the context of breast cancer, treatment with ephrinA1-Fc has been reported to attenuate epidermal growth factor-mediated phosphorylation of Erk and inhibit the transformation of NIH3T3 cells expressing v-erbB2 [57]. Another member of the Eph receptors, EphA2, has been linked to increased susceptibility to chemical carcinogen-induced skin cancer, as well as elevated tumor cell proliferation and phosphorylation of Erk when deficient in mice [58]. In neuroblastoma patients, the expression of EphB6, ephrin-B2, and ephrin-B3 in tumors has been associated with a positive prognosis. Additionally, ectopic expression of EphB6 has shown the ability to suppress the malignant phenotype of neuroblastoma cell lines [59]. A study on squamous cell carcinoma revealed a significant down-regulation of EphB1 by a factor of 1.1-fold and a similar down-regulation of ephrinB2 by a factor of 1.0-fold [60]. Moreover, the EphB4 receptor has been reported to function as a tumour suppressor in a mouse xenograft model of breast cancer. This effect is elicited when the receptor is stimulated by its ligand, ephrinB2 [13].

## The potential mechanisms of Eph function in tumor development

Although the role of Eph receptors in cancer development has been observed, the exact mechanisms underlying their oncogenic effects are still not fully understood [50]. Studies have shown that the overexpression of Eph receptors and ephrins is associated with angiogenesis and metastasis in various human cancers [17]. It has been hypothesized that these receptors may play a central role in angiogenesis or metastasis, rather than acting as primary oncogenes [45]. Ongoing research is dedicated to unraveling the complex cellular and molecular mechanisms involved in Eph-ephrin interactions and signaling. Our current focus is on understanding the potential mechanisms behind the dual roles of Eph receptors. Given their involvement in critical processes such as angiogenesis in tumor tissues, these molecules have the potential to serve as prognostic markers for tumors and show promise as targets for cancer therapy [17].

### Conflicting effects of Eph signaling

**Fig. 2:**
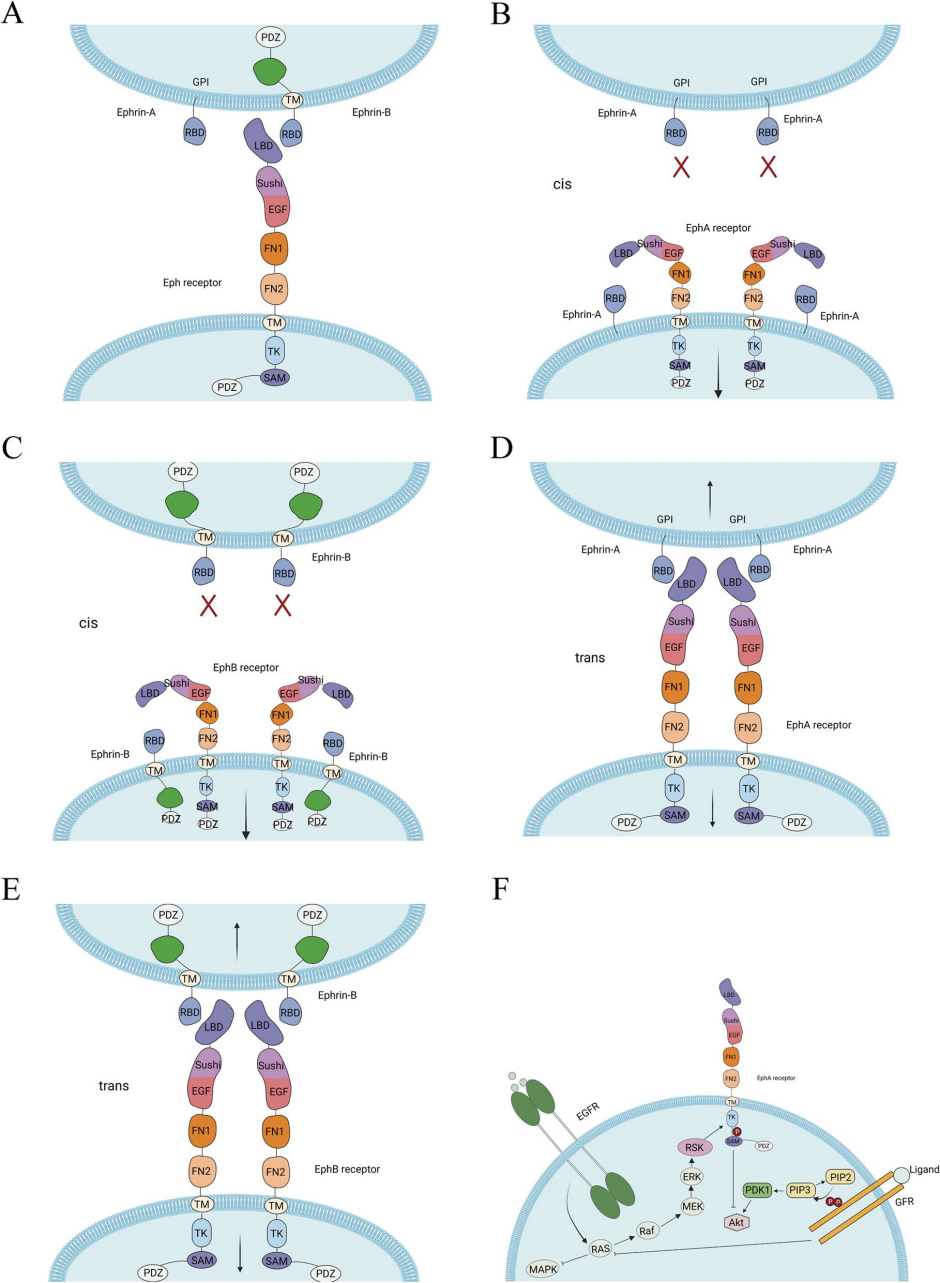
The molecular structure of Ephrin-Eph and signaling pathway. **A** The molecular structures of ephrinA and ephrinB are different, while their receptors are similar. **B**, **C** The EphA and EphB receptor can bind to the ligands present on the same cell with the receptor, known as cis signaling, activating downstream signals. **D**, **E** The EphA and EphB receptor can bind to ligands located on neighboring cells, referred to as trans signaling, activating downstream signals. **F** Ephrin-Eph signaling engages in crosstalk with other RTKs such as EGFR and GFR, involving ERK, Ras and other downstream molecules

The Eph receptor family share a common structural feature known as the RTK domain, which contains the catalytic kinase domain responsible for phosphorylation. The extracellular domain of Eph receptors includes an N-terminal ligand-binding domain (LBD), a cysteine-rich domain (CRD) with an epidermal growth factor (EGF)-like motif, and two fibronectin-type III repests. This is followed by a single-pass transmembrane domain and an intracellular segment consisting of a juxtamembrane region, a tyrosine kinase domain, a sterile α motif (SAM), and a PDZ (postsynaptic densityprotein PSD95, Drosophila disc large tumor suppressor DlgA, and zonula occludens-1 protein ZO-1) -binding motif [61, 62]. Both EphA and EphB receptors share similar structural elements, including the extracellular ligand-binding domain, the transmembrane domain, and intracellular domains that initiate signaling cascades (Fig. 2A). Activation of EphA or EphB receptors can be induced preferentially by ephrin-A or ephrin-B ligands, respectively, or through cross-activation. In addition to ligand-induced phosphorylation signals, ligand-independent receptor clustering can generate oligomeric assemblies and modulate transmembrane signaling [63]. Eph receptors can also signal in a ligand-independent manner, particularly when they are overexpressed, as observed in cancer cells [64]. Eph receptors and ephrin ligands can engage in cis-interactions when both the ligand and receptor are expressed in the same cells. The diverse signaling mechanisms add complexity to the Eph-ephrin system, contributing to its diverse function in tumorigenesis.

#### Forward and reverse signaling: tyrosine kinase-dependent pathways

**Fig. 3:**
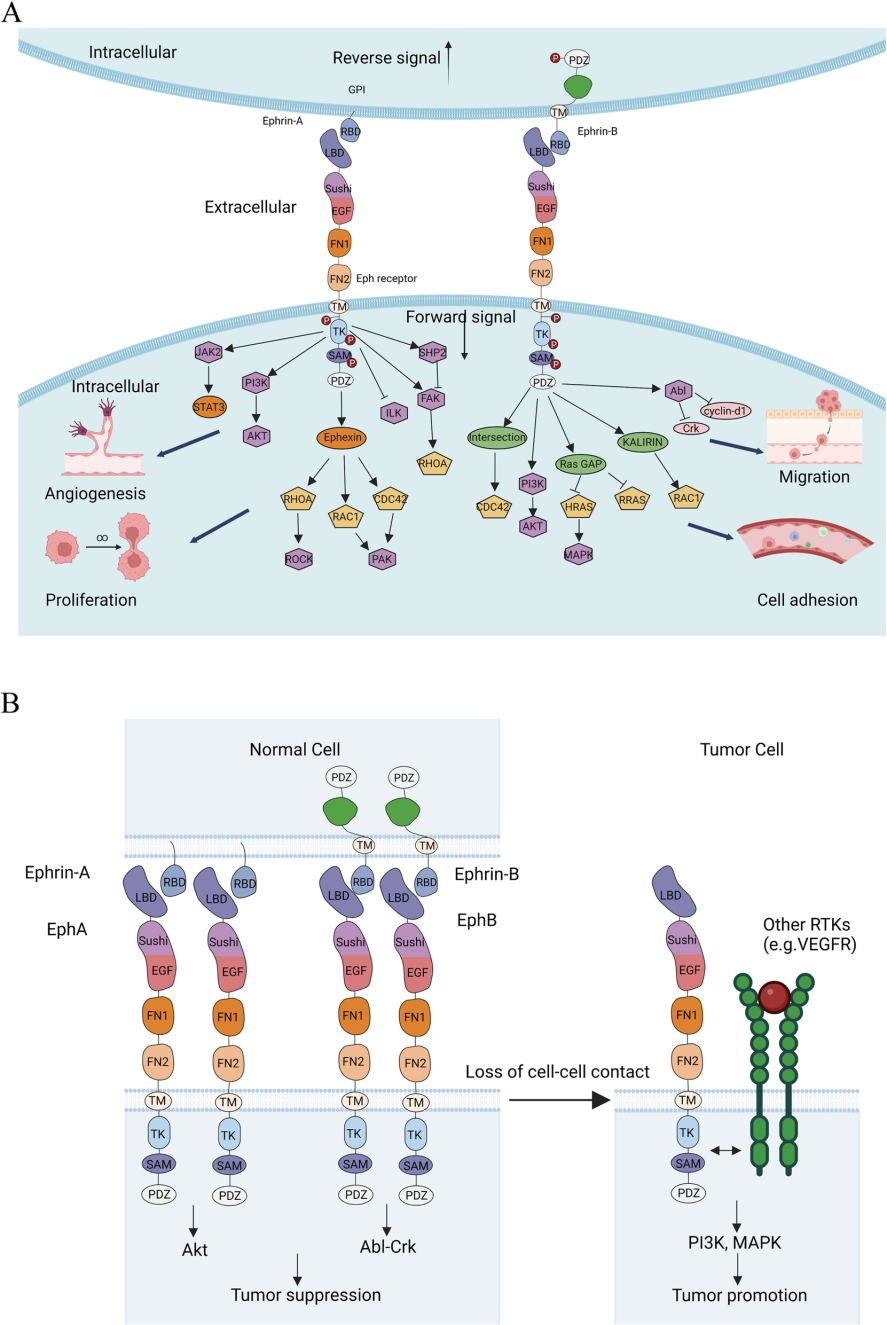
Working models of Eph receptors and their ligands in physiological functions. **A** Ephrin-Eph signaling contains reverse signal and forward signal, leading to the phosphorylation of downstream molecules. The forward signal influences the expression of additional downstream molecules, thereby impacting processes such as angiogenesis, cell proliferation, cell adhesion, and migration. **B** Ephrin-Eph signaling engages in cross talk with other RTKs, such as VEGFR, exerting regulatory effects that can either suppress or promote tumor development

Eph tyrosine kinases play a crucial role in a bidirectional signaling, involving forward signaling from ephrin-expressing cells to Eph receptor-expressing cells and reverse signaling from Eph receptor-expressing cells to ephrin-expressing cells [65]. Eph receptors mediate tyrosine kinase-dependent “forward” signaling, while ephrins, specifically ephrinB proteins, mediate ‘reverse’ signaling through phosphorylation by cytoplasmic tyrosine kinases [66] (Fig. 3A). Both types of signaling can lead to cell repulsion, depending on clustering intensity, downstream signaling strength, and Eph/ephrin internalization rates [67]. Eph-mediated forward and ephrin-mediated reverse signaling can be initiated through tyrosine phosphorylation of intracellular residues [68].

Forward signaling of Eph receptors plays significant roles in various physiological and pathological processes, including embryonic development, neuronal guidance, immune responses, and cancer progression. Mutations that impair kinase function of Eph receptors, such as EphB2, EphA3, EphA5, are frequently found in cancer, suggesting a tumor suppressor role for Eph receptor forward signaling [69–71] (Table 2). Eph forward signaling inhibits proliferation, survival, migration and invasion in several cancer types through regulation of oncogenic signaling pathways, including ERK, RAS/MAPK, PI3K-AKT, Abl-Crk [11, 13, 50, 61]. Stimulation of EphA2 by ephrinA1 inhibits Akt activation and subsequently induces dephosphorylation of EphA2 on S897 [11]. EphrinA1-induced stimulation of EphA2 also leads to reduced ERK phosphorylation and suppresses growth of primary keratinocytes and prostate carcinoma cells [50]. EphB4, in breast cancer cells, activates an anti-oncogenic Abl-Crk pathway, inhibiting cell viability, proliferation, motility, and invasion, and downregulates the pro-invasive matrix metalloprotease, MMP-2 [13].

**Table 2:** The mutation of Eph family

Eph receptors	Mutation site	Tumor type	Functions	References
EphA2	the first FNIII domain	lung squamous cell carcinoma	focal adhesions and actin cytoskeletal regulatory proteins	[72]
EphA3	copy number variations of EphA3	leukemia	lack forward EphA3 kinase signaling activities	[73]
EphA4	L920 to phenylalanine (L920F)	melanoma	potentiate EphA4 autophosphorylation and signaling	[74]
EphA5	missense mutations and truncation	lung adenocarcinoma	impair natural killer (NK) cell-mediated cytotoxicity, CD8 T cell exhaustion	[75]
EphA7	somatic missense mutations	aggressive leukaemic variant of cutaneous T-cell lymphoma	affect apoptosis and T-cell maturation	[76]
EphA8	lack the juxtamembrane portion	not mentioned	be defective for endocytosis with ephrinA5	[77]
EphA10	lack 3 critical motifs necessary for catalytic activity	not mentioned	the absence of kinase activity	[78]
EphB1	loss-of-function uORF mutationsthe uAUG to uGUG mutation	breast and colon cancer	induce enhanced translation of the downstream coding sequence to various extends	[79]
EphB2	D862N	colorectal tumor	promote TNF signaling activation	[80]
EphB3	substitution of both two tyrosine residues(JM) with glutamic acids	non-small-cell lung cancer	keep the receptor in an autoinhibited state	[81]
EphB4	two (G723S and A742V) in the tyrosine kinase domain; and one (P881S) in an intracellular linker region just carboxy-terminal to the tyrosine kinase domain	lung cancer	reduce phosphorylation of the A742V and P881S variants	[82]
EphB6	a missense mutation(Q926R)	lung cancer	reduce the flexibility of the SAM domain, suppress c-Cbl recruitment	[83]

Eph forward signaling plays a crucial role in regulating the actin cytoskeleton, which is essential for cell migration and axon guidance. EphB forward signaling is pivotal for filopodial motility and synapse formation, activating PAK, a serine/threonine kinase that governs actin dynamics [67]. Eph receptors also contribute to epithelial homeostasis maintainance and can induce mesenchymal-to-epithelial transition (MET) in cancer cells. For instance, EphA2, EphA4 and several EphB forward signaling pathways, including EphB3 and EphB2, are involved in promoting MET by enhancing the expression of E-cadherin [61, 84]. Examining EphA2 in more detail, its phosphorylated tyrosine kinase activity inhibits the chemotactic migration of cancer cells. Conversely, ligand-independent overexpression of EphA2 promotes cancer cell migration through AKT-induced serine phosphorylation [11]. Importantly, EphrinA1 stimulation of EphA2 disrupts the interaction with AKT, leading to the dephosphorylation on S897 on EphA2. This, in turn, blocks the effects on migration and cell invasion [11]. Furthermore, the upregulation of EphA3 inhibits phosphorylation of PI3K/STAT3/AKT, enhancing apoptosis and reversing cellular resistance to cisplatin [85, 86].

Eph reverse signaling, initiated by ephrins within host cells, adds complexity to the Eph-ephrin system. Upon engagement with Eph receptors in neighboring cells, ephrins activate their cytoplasmic tails, recruiting and activating various intracellular signaling components, such as Src family kinases, ultimately influencing cellular responses like morphological changes, motility, and differentiation. The ephrinB cytoplasmic tail recruits and activates Src family kinases, resulting in tyrosine and serine phosphorylation and the recruitment of signaling effectors [67]. In vivo observations have highlighted the significance of ephrinB2-PDZ interaction for reverse signaling in lymphatic vessel development [87]. EphrinB2 reverse signaling triggers substantial morphological changes and increased motility [56], aligning with previous findings demonstrating that ephrin-B2 reverse signaling increases the motility of glioma cells [88]. During osteoclast differentiation, ephrinB2 expression is evident, and reverse signaling through ephrinB2 inhibits osteoclast differentiation by suppressing transcription factors Fos and NFATc1 [89].

In various cancer types, soluble Fc fusion proteins of ephrin ligands activate Eph forward signaling, resulting in decreased proliferation, survival, migration and invasion of various cancer cells in culture, also inhibiting tumor growth in several mouse models [13, 61]. The apparent paradox of Eph receptors being highly expressed but poorly activated by ephrins, as evidenced by their low level of tyrosine phosphorylation, suggests that Eph forward signaling may even be detrimental to tumor progression [90].

#### Eph cis- and trans- signaling: opposing functions

Eph receptors and ephrins engage in an intricate interplay involving both cis-interactions (within the same cell) (Fig. 2B and C) and trans-interactions (between neighboring cells) (Fig. 2D and E). Cis-interactions typically occur when both the ligand and receptor are expressed in the same cells [91]. Notably, cis-interactions do not activate Eph receptor tyrosine kinase activity. Instead, they diminish the receptor’s interaction with ephrin on adjacent cells, thereby dampening kinase activity [92]. The inhibition of cis-signal to trans-signal adds complexity to Eph receptor signaling and has been observed in various immune cell types, including NK cells, DC cells, mast cells, and T cells [93–95]. Plexin receptors and Notch receptors are also influenced by cis-binding [96]. The cell’s response to ligand-receptor interactions varies depending on expression levels and cell–cell contact. Tetramers of receptors and ligands are essential for both cis- and trans- signaling. The higher the number of cell surface receptors, the more likely they are to interact with ligands expressed on the same cells [96].

To explore the role of genes in tumorigenesis, researchers commonly employ gain-of-function or loss-of-function methodologies. In brief, introducing Eph receptors or ephrin ligand through transfection results in reduced kinase activity [97], leading to enhanced migration and invasion of cancer cells [41]. Conversely, using siRNA against Eph receptors leads to decreased migration and invasion [41]. For instance, Knockdown of EphA7 suppresses proliferation and metastasis in A549 human lung cancer cells [98]. Knockdown of EphB1 in medullobalstomas cells significantly reduces migration, which correlates with decreased β1-integrin expression and phosphorylated Src [99]. It is worth noting that Eph phosphorylative activity can suppress the progression of colorectal cancer [100]. Ligand-activated EphB1 signaling inhibits cancer cell migration and invasion through inducing EphB1 phosphorylation. Cis-interactions could be a strategy adopted by cancer cells to evade the tumor suppressive effects of Eph receptor signaling induced by trans-binding of ephrins [81].Upon tumor initiation, Eph receptor expression is upregulated through oncogenic signaling pathways [57]. However, the ephrin ligands often undergo down-regulation or are unable to bind to receptors due to the loss of cell adhesion [101]. Eph receptors require activation by ligands attached to the surface of neighboring cells, indicating that stable cell–cell adhesion may be necessary for Eph activation [101]. Another study suggests that impaired cell–cell adhesion in tumor cells hinders the activation of Eph receptors by ephrins located on adjacent cells [50].

In some cases, tumors exhibit EphA2 overexpression, often associated with the loss of corresponding ligands [102]. Eph receptors and co-expressed ligands engage in direct cis interactions, devoid of initiating intracellular signals. Nevertheless, this cis interaction hinders trans-interactions [92]. Similarly, coexpressed ephrin ligands with Eph receptor attenuate Eph receptor kinase activation [103]. Cis-interactions are ligand-independent, rendering Eph receptors less responsive to Ephrin ligands [6]. Trans-interactions, on the other hand, are ligand-dependent and rely on specific ephrins for Eph receptor functionality. Despite high expression in cancers, Eph recetors within cancer cells are often poorly tyrosine-phosphorylated [104].

The reversible process of epithelial-mesenchymal transtion (EMT) and mesenchymal-epithelial transtion (MET) further illustrates this complexity. In EMT cells, EphB1 cis-signaling counteracts trans-signaling due to reduced cell–cell contact [105]. However, in the MET state, where cell–cell adhesion is regained, EphB1 and ligand ephrinB2 interactions in trans promote cancer cell stemness, affecting genes like Nanog and Sox2. Cancer cells acquire stem cell characteristics and resistant to chemotherapy via context-dependent EphB1 signaling.

#### Ligand-dependent- and -independent signaling

Eph receptors have the ability to form clusters through direct Eph-Eph interactions, even in the absence of Ephrin ligands [62]. This clustering is facilitated by the ligand-binding domain (LBD) and cysteine-rich domain (CRD), both of which play a crucial role in promoting Eph-Eph interaction [62, 65] . Studies have indicated that the FN, CRD and LBD domains are pivotal in Eph signaling, allowing them to form tetrameric complexes independently of ephrins. It’s noteworthy that the FN domain may also be significant for EphB1 binding with ephrin ligands other than EphrinB2 [105].

Eph receptors and their ligands, ephrins, engage in a complex interplay of both ligand-dependent and ligand-independent signaling. Ligand-dependent signaling occurs when Eph receptors interact with their ligands (ephrins) on adjacent cells, initiating a series of events, including receptor clustering and subsequent phosphorylation. Conversely, ligand-independent signaling involves Eph receptors functioning independently of ephrin engagement. In cases where Eph receptors exhibit low levels of phosphorylation in cancer cells, their tumor-promoting activities are likely to be independent of Ephrin stimulation [61]. Factors such as increased phosphotyrosine phosphatases activity or the loss of E-cadherin in tumor cells can lead to under-phosphorylation of Eph receptors, despite their overexpression [50]. Loss of cell–cell contacts can also prevent ligand stimulation from neighouring cells, reducing Eph receptor phosphorylation [105]. E-cadherin plays a role in regulating the cell surface localization of EphA2 in breast cancer cells, and the loss of cell–cell contacts can impede interaction with ephrin ligands [101], making Eph receptor oncogenic activity appear to be ligand independent [50]. Upregulation of Eph receptors can facilitate Eph receptor-mediated signaling through dimerization and higher-order oligomerisation, independent of ephrin ligation, and involving intracellular SAM domain and extracellular LBD-LBD, CRD-CRD interface [62]. Ligand-independent Eph receptor signaling is distinct from, and sometimes even opposite to, ligand-induced RTK activity [62].

Ligand-independent Eph receptor signaling operates through various mechanisms. Besides tyrosine residues, Eph receptors can also undergo phosphorylation on serine/threonine residues, resulting in functional consequences [11]. For instance, ligand-independent serine phosphorylation of EphA2 stimulates migration, invasion, and cancer progression, particularly when ligand-induced forward signaling is decreased [11]. Phosphorylation of serine/threonine residues in Eph receptors can trigger ligand-independent signalings, as exemplified by S897-phosphorylated EphA2, which activates AKT and PI3K, promoting the migration and invasion of cancer cells. A mutation at the S897 site abolishes the ligand-independent promotion of cell motility [11, 106]. Ligand-independent EphB1 signaling promotes lung cancer cell invasion and migration through CDH2-induced EMT [41]. Interestingly, tyrosine phosphorylation of Eph receptors induced by ligands can lead to dephosphorylation of S897, and multiple studies have confirmed the pro-tumorigenic roles of ligand-independent Eph receptors [64].

#### Cross-talk with other signaling pathways

Eph receptors can also impact other signaling pathways by physically interacting with other RTKs [50]. This crosstalk between Eph receptors and other RTKs, such as ErbB2 (HER-2) and EGFR, leads to the phosphorylation of Eph receptor tyrosine kinase, subsequently increasing the activation Rho GTPases and Ras/ERK signaling pathways [5, 51] (Figs. 2F and 3B). This interplay between Eph receptors and other RTKs, possibly in an ephrin-independent manner, promotes pro-oncogenic effects, including enhanced cancer cell motility and proliferation [50].

In summary, when ligand ephrins are present, Eph receptor forward signaling and reverse signaling counteract pro-oncogenic effects. However, in the absence of ligand ephrins, Eph receptor ligand-independent signaling promotes the pro-oncogenic effects. The physical interactions between ephrin and Eph receptors within the same cell, as well as the interaction of Eph receptor with other RTKs, contribute to processes such as proliferation, differentiation, adhesion and migration [107].

### Role of Eph and ephrin in vascular system

Tumor angiogenesis plays a crucial role in promoting cancer cell growth and metastasis. The human vascular system comprises a complex network of blood vessels, including arteries, veins, and capillaries, acting as the primary conduit for information exchange and metabolic substance transport [108]. This intricate vascular network facilitates the absorption of essential nutrients and the removal of cellular and metabolic waste products [109]. During early embryonic development, the vascular network emerges through vasculogenesis and angiogenesis. In adulthood, under physiologic conditions, blood vessels generally remain quiescent, with limited new branch formation [110].

There are three main mechanisms of vessel formation. First, vasculogenesis involves the de novo generation new blood vessels from endothelial progenitor cells [110]. Initially thought to be restricted to embryonic development, recent studies have reported instances of vasculogenesis occurring in adult tissues [111]. Second, sprouting angiogenesis is the process of forming new blood vessels from preexisting ones and is observed during wound healing, ovulation, and embryo development [112]. This highly invasive process relies on the regulation of endothelial cell proliferation and migration by proangiogenic and anti-angiogenic molecules [113]. Third, intussusceptive angiogenesis is characterized by the formation of intraluminal tissue pillars within existing capillaries, small arteries, and veins, resulting in the splitting of vessels into two daughter vessels through the development of a central perforation and subsequent fusion of multiple capillaries [114].

The role of angiogenesis in cancer progression has gained considerable attention, as it sustains tumor growth, invasion, and metastasis. Tumors often stimulate the development of new blood vessels by secreting pro-angiogenic factors like vascular endothelial growth factor (VEGF) and fibroblast growth factor (FGF), while anti-angiogenic factors such as thrombospondin-1, angiostatin, and endostatin act as negative regulators of angiogenesis.

**Fig. 4:**
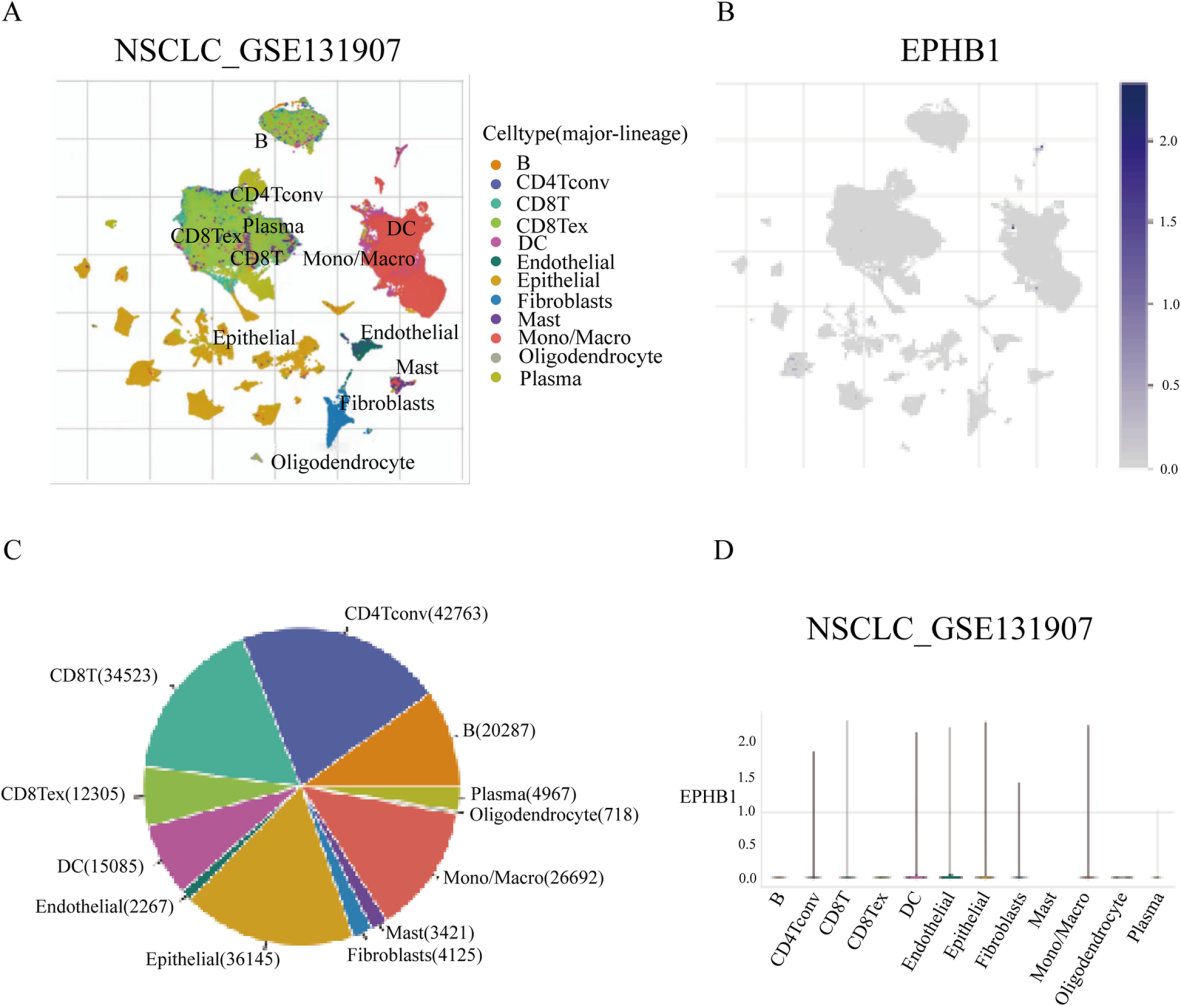
The expression of EphB1 in various cell types. The expression of EphB1 in various cell types was analyzed in publicly accessible single-cell RNA-seq (scRNA seq) dataset of colorectal cancer tissues (EMTAB8107). **A** UMAP plot with cell type annotations displayed at the top of the tab. **B** UMAP plot shows the expression level of EphB1 in different cell types. **C** Pie plot shows the cell number distribution of each cell type. **D** Violin plot reflects the distribution of EphB1 gene expression in various cell types

**Fig. 5:**
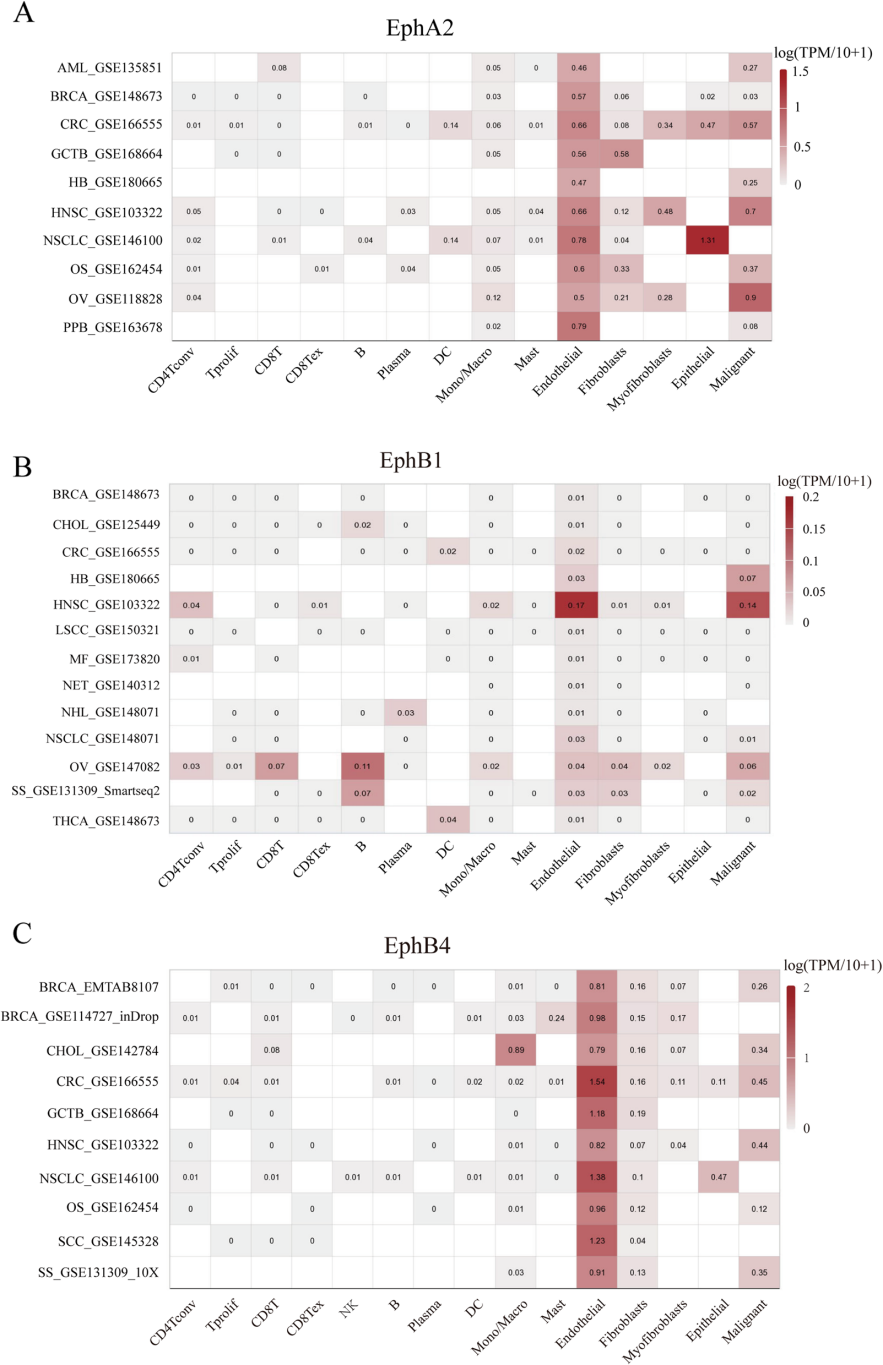
Average expression of Eph receptors. The TISCH2 database (http://tisch.compgenomics.org/), containing scRNA-seq data from more than 50 cancer types, was utilized to analyze the expression of Eph receptors. **A** The expression of EphA2 across datasets in different cell types. **B** The expression of EphB1 across datasets in different cell types. **C** The expression of EphB4 across datasets in different cell types

Eph receptor tyrosine kinases play pivotal roles in angiogenic processes, particularly in directing angiogenic sprouting and intussusceptive angiogenesis (Table 3). Several B-class ephrins and their receptors, including EphB2, EphB3, EphB4, ephrin-B1 and ephrinB2, are expressed in the murine embryonic vasculature [44]. Ephrin-B2, for example, is predominantly expressed in arteries and absent from veins [115], suggesting its role in defining boundaries between veins and arteries and influencing the location of capillary beds [44]. EphB2 and ephrinB2 are often located in the mesenchyme surrounding vessels, indicating their involvement in mesenchymal-endothelial interactions [44]. Genetic studies in mice have shown that targeted mutagenesis of ephrinB2 results in significant disruptions in normal vascular development during embryogenesis [116]. EphA2 activation induces a PI3-kinase-dependent signaling pathway that leads to the activation of Rac1 in endothelial cells in response to ephrin-A1. Analyzing single-cell sequencing data of colorectal cancer revealed endothelial cells as a prominent cluster, expressing the EphB1 gene significantly (Fig. 4). Moreover, similar analyses across 13 different tumors demonstrated substantial EphB1 expression in endothelial cells (Fig. 5A). EphA2 and EphB4, the most studied Eph molecules, were also found to be highly expressed in endothelial cells based on single-cell sequencing data (Fig. 5B and C). This signaling cascade orchestrated by Eph receptors contributes to vascular assembly and cell migration, further highlighting the crucial role in angiogenesis [117].

**Table 3:** The Role of Eph and ephrin in vascular system and immunity

	System	Type	Eph member	Mechanism
Role of Eph and ephrin	Vascular system	Sprouting angiogenesis	EphA2	Regulating endothelail cell assembly and migration by PI3K-mediated activation of Rac1GTPase
			EphB1	Recruit c-Src and induce its active conformation
			EphB4	Play a regulatory role in monocyte extravasation
		Non-sprouting intussusceptive angiogenesis	EphB4	PDGF-β, MCP-1 and Eph/ephrins are potential candidates for mediating IA. ERK1/2 activity is required for EphB4 regulation of VEGF-induced intussusceptive angiogenesis
	Immune modulation and inflammation	Immune Cell Differentiation	EphB4	The interation between EphB4 and ephrin-B2 governs the mobilization of hematopoietic stem and progenitor cells from bone marrow to the circulation
		Immune Cell Activation	EphA2	DCs loaded with EphA2 peptide induce immune responses and reduce tumor burden
			EphA3	Suppress tumor through suppressing ATK activation
			EphB2	Prior to activation, upregulation of EphB2 leads to increased proliferation and antibody production in human B cells
			EphB4	Inhibit T cell proliferation by inducing the production of immunosuppressive factors
		Inflammation	EphB1	Particularly through STAT3, promote protective, anti-inflammatory, or immunomodulatory pathways

#### Role of Eph and ephrin in sprouting angiogenesis

Sprouting angiogenesis plays a fundamental role in the development of new blood vessels in cancer. This process involves the emergence of new capillaries or blood vessels branching out from existing ones, premarily driven by signals such as Vascular Endothelial Growth Factor (VEGF). In this intricate process, Eph receptors and their corresponding ephrin ligands play crucial roles as regulators, influencing various aspects of angiogenic sprouting, including endothelial cell migration, adhesion, proliferation, and vessel branching.

It’s important to note that Eph/ephrin signaling doesn’t directly induce cellular proliferation but rather exerts its effects through the involvement of other growth factors [118]. For instance, EphB/ephrinB signaling triggers Akt, PI3K, and MAPK signaling pathways, promoting endothelial cell proliferation and migration [29]. EphA2/ephrinA1 signaling regulates endothelail cell assembly and migration by PI3K-mediated activation of Rac1GTPase [119]. Inhibition of EphA receptors using soluble EphA-Fc or EphA3-Fc has been shown to effectively hinder tumor angiogenesis [120]. The role of Src as a key mediator in specific angiogenic processes has been highlighted [121]. It has been found that c-Src is an important target of EphB1. Ligand-stimulated EphB1 recruits c-Src and induces its active conformation through the phosphorylation of tyrosine Y418 [122].

Among the extensively studied Eph-ephrin pairs, EphB4 and ephrinB2 not only promotes the sprouting of new blood vessels but also play a crucial role in vessel maturation. EphB4 is primarily expressed in venous endothelial cells, while ephrinB2 is predominantly found in arteries. Their interaction facilitates endothelial cell migration, sprouting, and the assembly of new vessels. Additionally, interaction between endothelial ephrin-B2 and monocytes EphB4 plays a regulatory role in monocyte extravasation through the vascular endothelium [123, 124]. The analogous process of cancer cell extravasation driven by ephrinB2-EphB4 is yet to be determined [61].

#### Role of EphB and EphrinB2 in non-sprouting intussusceptive angiogenesis

Intussusceptive angiogenesis (IA) plays a significant role in both pre- and postnatal vascular growth,remodeling, tissue repair, and cancer progression [125]. Unlike sprouting angiogenesis, which relies on endothelial cell proliferation and migration, IA is a non-sprouting mechanism involving the division or splitting of pre-existing blood vessels. This distinctive process facilitates the expansion of the capillary bed and the development of intricate vascular networks while maintaining the architectural integrity of the tissue [125]. IA is a rapid process that does not require endothelial cell proliferation.

The mechanisms governing IA involve a complex interplay of hemodynamic forces, endothelial cell behavior, and extracellular matrix remodeling. Elevated fluid shear stress and mechanical forces within blood vessels are thought to initiate the formation of transvascular tissue pillars, leading to the division of the vessel lumen into multiple channels. Subsequently, these tissue pillars undergo growth and fusion, giving rise to new vessel segments and expanding the vascular network.

Several factors that are involved in sprouting angiogenesis, such as PDGF-B, MCP-1 and Eph/ephrins, are potential candidates for mediating IA [125]. Eph/ephrin signaling pathways, in particular, play a crucial role in regulating the positioning and segregation of arterial and venous endothelial cells during angiogenesis [116].

EphB4/ephrinB2 regulates IA in response to VEGF [126]. EphB4 in endothelial cells finely tunes the extent of endothelial proliferation induced by VEGF, thereby controlling the initial vascular enlargement without directly affecting VEGF-R2 activation [126]. Instead, it modulates downstream signaling through MAPK/ERK. Blocking ephrinB2/EphB4 signaling, without affecting TGF-β1/TGF-βR or angiopoietin/Tie2 pathways, can shift VEGF-induced angiogenesis from normal to aberrant. Conversly, activation of EphB4 signaling prevents the occurrence of aberrant angiogenesis induced by high VEGF doses.

### The role of the Eph family in immune modulation and inflammation

Eph receptors play a multifaceted role in immune cell differentiation and activation (Table 3). These receptors are expressed widely in immune cells, such as monocytes, macrophages, DCs, B cells and T cells. They contribute to diverse functions within the immune system, encompassing immune cell activation, migration, adhesion, differentiation and proliferation, often modulated by interactions with the microenvironment [127].

#### Immune cell differentiation

Eph receptors play roles on hematopoietic cells during their differentiation into distinct immune cell fates [127]. The cell fate decisions are critically dependent on the interaction of the hematopoietic stem cells with their microenvironmental components [127]. The interplay between EphB4-positive human bone marrow hematopoietc progenitor cells and ephrin-B2-positive stromal cells significantly influences the differentiation of progenitor cells into mature erythroid cells [128]. Additionally, the interaction between EphB4 and ephrin-B2 governs the mobilization of hematopoietic stem and progenitor cells from the bone marrow to the circulation [129]. In cellular immunity, T cells play a crucial role and can be stimulated by ephrin-B1 and ephrin-B2, which regulate thymocyte development [130]. Deletion of ephrin-B1 and/or ephrin-B2 in thymocytes or thymic epithelial cells (TECs) results in decreased medullary areas and enlarged cysts [131].

#### Immune cell activation

Immune cell activation is a critical mechanism employed by the body to defend itself against infections and cancers. This intricate process involves antigen recognition, antigen presentation, activation signals, co-stimulation, and eventual immune cell activation. Upon activation, immune cells initiate a series of events, including the production of cytokines, chemokines, and antibodies, along with increased cell proliferation and recruitment to the site of infection, inflammation and cancer. While the immune system’s role in recognizing and eliminating cancer cells is pivotal, cancer cells can employ strategies to evade immune surveillance and suppression. The regulatory influence of Eph receptors extends to immune cell activation, influencing their capacity to detect and target tumor cells. This regulatory role can lead to either heightened or diminished anti-tumor immune responses, thereby adding complexity to the relationship between immune cell activation and cancer.

EphA3 is frequently overexpressed in various types of tumors, such as melanoma, lung carcinoma, and sarcoma, where it functions as a tumor-specific antigen [132]. Despite its oncogenic roles in gastric carcinoma and glioblastoma multiforme [133, 134], EphA3 may function as a tumor-suppressor in lung adenocarcinomas through suppressing ATK activation [86]. EphA3 is also the most highly mutated Eph receptor, and many EphA3 mutations identified in cancers impair kinase activity. This suggests that wild-type EphA3 may inhibit cancer formation or progression, and somatic cancer mutations disrupt these tumor-suppressive activities [86]. EphA3 mutations are particularly frequent in lung cancer and can act as “drivers” in lung cancer [135]. Dendritic cells (DCs) capture, process, and present antigens to other immune cells, such as T cells and B cells. DCs loaded with EphA2 peptide have been found to induce immune responses and reduce tumor burden [136, 137]. In T cells, EphB receptors, including EphB1, EphB2,EphB3 and EphB6, are expressed [127]. In vitro assays have shown that stimulation by ephrin B ligands can activate T cells, but high concentration of ephrinB1 and ephrinB2 can inhibit T-cell activation, suggesting that EphB-ephrinB signaling may also provide negative feedback during T cell activation [127, 138–140]. In a rat glioma model, ephrin-A1 can activate DCs [141]. EphB1 is expressed in plasmacytoid DCs, CD4^+^ and CD8^+^ T cells [127, 142]. EphB6, primarily expressed in thymocytes and a subpopulation of T cells, plays a crucial role in T cell activation and cancer cell death. The absence of EphB6 in mice reduces T cell activation and the recruitment of crucial signaling molecules like ZAP-70, LAT, SLP-76, PLCγ1, and P44/42 MAPK [143]. The compensatory functions within the ephrin family are evident, as the knockout of ephrin-B1 or ephrin-B2 in mice does not affect the activation and proliferation of T cells and native CD4 cells [130]. Notably, in human bone marrow-derived mesenchymal stromal cells, ephrinB2 binding to EphB4 on T cells inhibits T cell proliferation by inducing the production of immunosuppressive factors and reducing activating cytokines [140]. These findings highlight the regulatory roles of Eph receptors and ephrins in immune cell activation and their potential implications in anti-tumor immune responses.

The peripheral B cell pool becomes populated with antigen-experienced memory cells, which is similar to the T pool. The activation and antibody production of these B cells require interactions with other immune cells. B cells are primarily responsible for orchestrating humoral immunity [144]. The expression levels of Eph receptors and ephrin ligands vary between activated and non-activated B cells [145]. This variation suggests that they may play a role in processes facilitating B cell activation. Research has shown that prior to activation, the upregulation of EphB2 leads to increased proliferation and antibody production in human B cells [127]. The differential expressions of Eph receptors and ephrin ligands in B cells not only regulate their development, activation, differentiation, and functionality but also potentially contribute to their specialize into various canonical B cell fates [127].

#### Inflammation and inflammatory pain

Eph receptors have gained increasing recognition for their significant roles in inflammatory diseases such as rheumatoid arthritis and bone cancer pain. They play a crucial role in regulating the migration and adhesion of leukocyte, contributing to the regulation of inflammatory signaling pathways. Eph receptors and ephrins also participate in tissue repair and remodeling following inflammation.

EphB1, for example, has been identified as beneficial to the activation of STAT3 transcripts and networks, suggesting a shift from proinflammatory activation towards an immune-modulatory or anti-inflammatory arm of the STAT3 pathway [146]. EphrinB1 and EphB1 exert significant influence on T cell function, playing crucial roles in inflammatory conditions like rheumatoid arthritis [147]. EphrinB1 can activate EphB1, leading to the production of TNF-α in peripheral blood lymphocytes and IL-6 in synovial cells [147]. Metastatic cancer-induced bone pain (CIBP) is a complex chronic condition [148]. Inflammation contributes to CIBP, and anti-inflammatory drugs such as nonsteroidal anti-inflammatory drugs (NSAIDs) are commonly used for treatment [149]. EphrinB-EphB receptor signaling, significantly upregulated in the dorsal root ganglion and spinal cord, has been implicated in the development of bone cancer pain [150]. Studies demonstrate that intrathecal injection of ephrinB2-Fc induces thermal hyperalgesia, mechanical allodynia, and activates spinal PKA and CREB. Inhibition of PKA can prevent and reverse thermal hyperalgesia and mechanical allodynia caused by ephrinB2-fc [151]. The involvement of EphB1 forward signaling in the spinal cord has been linked to the development of bone cancer pain and morphine tolerance. Agents designed to block the EphB1 receptor, such as EphB2-Fc, have demonstrated the potential to prevent and reverses bone cancer-induced pain [150, 152]. EphB1-Fc not only inhibits the activation of spinal MAPKs induced by inflammatory and neuropathic pain but also potentially hinders the formation of long-term potentiation (LTP) at synapses between dorsal root gnaglia and dorsal horn [153]. Furthermore, EphrinB-EphB receptor signaling activates astrocytes and microglial cells in the spinal cord by either activating or interacting with TLR4. This activation results in increased activity of proinflammatory cytokines like IL-1B and TNF-ɑ, contributing to bone cancer pain [154]. In another aspects, EphA4 receptor is involved in the generation and maintenance of CFA-induced chronic inflammatory pain, and blocking the spinal EphA4 receptor could potentially relieve persistent pain behaviors in mice [155].

## Therapeutic implications

The intricate nature of Eph signaling presents a challenge for targeted cancer treatment. Various strategies exist to either enhance or inhibit the activities of individual Eph receptor or multiple family members. Despite the potential of these strategies, there are currently a lack of approved drugs specifically targeting the Eph family for cancer therapy.

### Monoclonal antibody

**Fig. 6:**
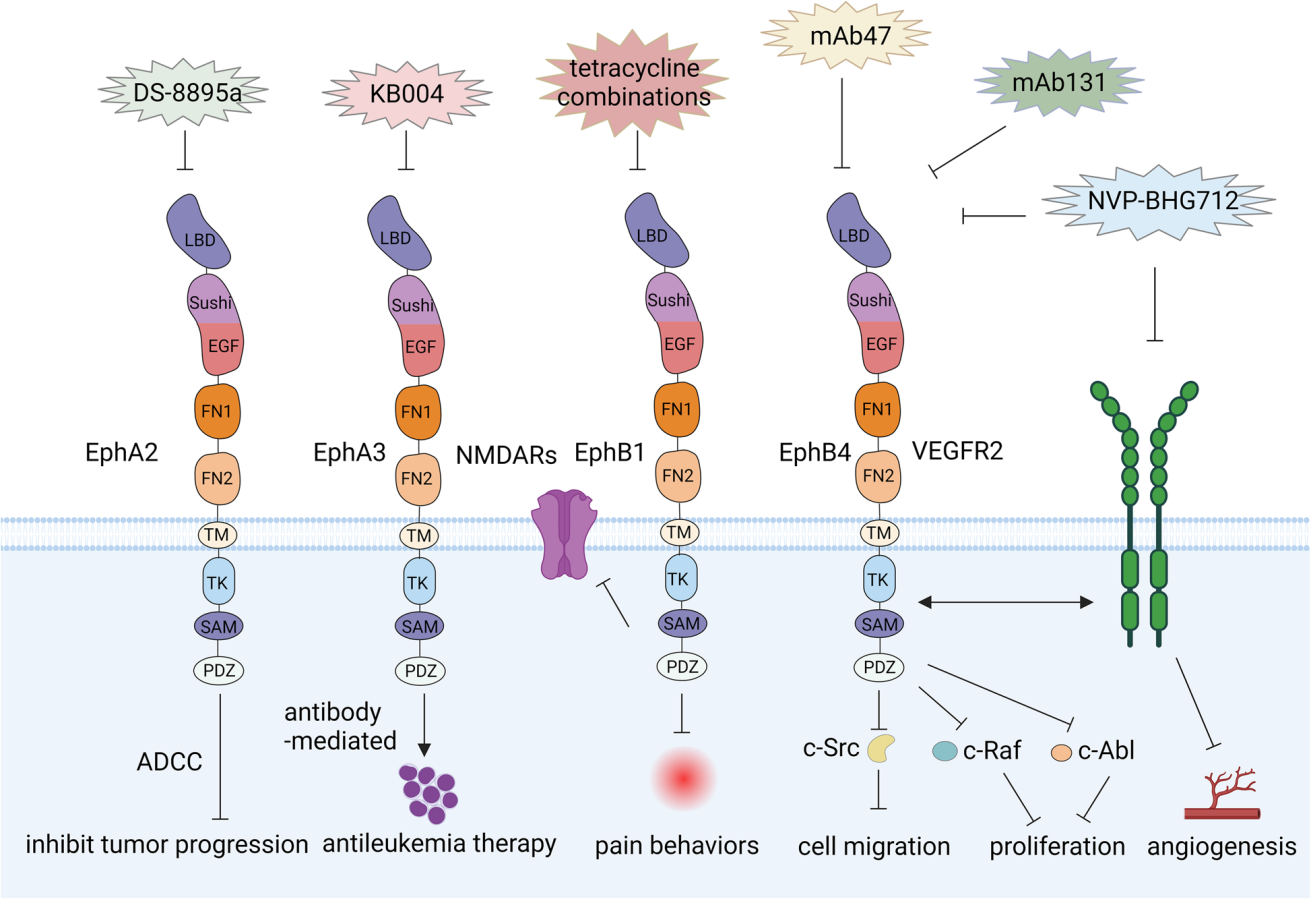
Therapeutic strategies targeting Eph receptors. These strategies can inhibit tumor progression through various approaches and also impact pain behaviors

Monoclonal antibody drugs represent a targeted therapy utilizing antibodies to specifically bind to certain proteins on the cell surface. This approach offers several advantages for the development of Eph-targeted therapeutics: (1) Monoclonal antibody drugs can be precisely designed to target specific proteins or cells, minimizing the risk of damage to healthy cells and tissues; (2) Monoclonal antibody drugs function as agonists for receptors by inducing receptor clustering, leading to activation. They can also act as antagonists by reducing receptor levels through processes like receptor endocytosis and degradation; (3) Monoclonal antibody drugs can inhibit receptor activity by blocking ligand binding, disrupting downstream signaling pathways; (4) Monoclonal antibody drugs can exert cytotoxic effects directly or indirectly through mechanisms such as antibody-dependent cell-mediated cytotoxicity (ADCC) or complement-dependent cytotoxicity (CDC) (Fig. 6); (5) Monoclonal antibody drugs can be loaded with various payloads, including radioactive isotype, conjugated drugs, or durg-containing nanoparticles [156]. For instance, anti-EphA2 mAbs have developed as agonistic antibodies, effectively inhibiting tumor growth and angiogenesis by inducing receptor phosphorylation and subsequent anticancer effects [157]. Humanized EphA2 mAb DS-8895a has demonstrated effecacy in inhibiting the growth of breast and gastric xenograft models through ADCC [158]. Additionally, EphA3, often overexpressed in hematologic malignancies, has been targeted by the humaneered derivative of the IIIA4 anti-EphA3 mAb (KB004) in clinical trials for hematological malignancies. KB004 exhibits a direct and antibody-mediated antileukemic effect on EphA3-positive human pre-B-ALL xenografts, specifically targeting leukemic cells, and its payload with an isotope is highly effective for antileukemia therapy [159].

Among the EphBs, EphB4 has emerged as a prominent target for antibodies [156]. Disrupting the binding between ephrinB2 and EphB4 represents a novel strategy for anti-angiogenic therapy. Currently, two antibodies, including mAb47 and mAb131, have been developed to target the fibronectin domain of EphB4. Both mAbs have demonstrated the ability to reduce blood vessel density. Specifically, monoclonal antibody mAb47 has shown efficacy in suppressing tumor angiogenesis and inhibiting the growth of both EphB4-positive and EphB4-negative tumors, suggesting its impact on the tumor microenvironment [160, 161].

### Targeting serine phosphorylation of Eph receptors

#### Targeting tyrosine phosphorylation of Eph receptors

Targeting tyrosine phosphorylation of Eph receptors involves strategies aimed at inhibiting or modulating the activity of these cell surface receptors, particularly RTKs. Targeting RTKs can be achieved through different approaches, such as small molecule inhibitors or monoclonal antibodies. Small molecule inhibitors typically bind to the ATP-binding site of the kinase domain, thereby preventing the phosphorylation of the receptor. An example is NVP-BHG712, which serves as an EphB4 kinase inhibitor. Treatment with NVP-BHG712 leads to a dose-dependent inhibition of RTK phosphorylation. It also shows activity against EphA2, VEGFR2, c-Raf, c-Src and c-Abl. By inhibiting VEGFR, NVP-BHG712 disrupts the growth and survival of cancer cells, leading to tumor regression. NVP-BHG712 exhibits excellent pharmacokinetic properties, inhibits EphB4 autophosphorylation after oral administration, and effectively suppresses VEGF-driven vessel formation (Fig. 6). The data suggests a close relationship between VEGFR and Eph receptors signaling during vessel formation, and EphB4 forward signaling is a significant mediator of VEGF-induced angiogenesis [162]. Despite its potential as an anti-cancer agent, further research is needed to fully understand its mechanisms of action and determine its optimal use in different cancer types and patient populations.

Interestingly, EphB1 receptor forward signaling is critical to the development and maintenance of pain, involving the activation of NR1 and NR2B receptors and subsequent Ca^2+^ -dependent signals. Inhibition of EphB1 prevents pain behaviors and NMDAR activation, even in the presence of active ephrinB2. Conversely, activation of EphB1 receptor induces pain behaviors and NMDAR activation, even with down-regulated ephrinB2 [150] (Fig. 6). The activated Eph receptors can also facilitate other protein–protein interactions via SAM and PDZ-binding motifs, contributing to signaling [163]. Investigating the roles of Eph receptors in neuropathic pain, researchers have identified tetracycline combinations that exhibit inhibitory activity against EphB1 tyrosine kinase through in silico screen in FDA-approved drugs. These combinations hold the potential for modulating the EphB1 pathway, which is relevant to neuropathic pain [164].

### siRNA

Small interfering RNAs (siRNAs) approach serves as a valuable method to target the overexpression of mRNA. Inhibiting Eph receptors through siRNAs effectively disrupts Eph forward signaling, reverse signaling and even non-canonical signaling pathway [165]. Downregulation of EphA2 or EphB4 using small interfering RNAs (siRNAs) or antisense oligonucleotides has demonstrated significant impact on cancer cell malignancy in culture and has shown efficacy in inhibiting tumor growth in various mouse cancer models [61]. The knockdown of EphA2 in cancer cells in vitro has exhibited significant effects in glioma, NSCLC and breast cancer cells [166]. Furthermore, in vivo delivery of EphA2 siRNA using nanoliposomal in ovarian cancer has demonstrated effective targeting of cancer cell and remarkable anticancer effects [167, 168]. The knockdown of EphB4 in breast cancer cells has been shown to inhibit integrin-mediated cell adhesion, spreading and migration. However these effects are independent of ephrin stimulation [51].

## Conclusions and perspectives

Ephs and Ephrins have been the focal points of extensive research over several decades, illuminating their classification, structure, bidirectional signaling mechanisms. The comprehensive exploration of Eph receptors have unraveled their diverse roles across various biological processes, from embryonic development to cancer progression, offering valuable insights into the intricate landscape of cellular signaling networks.

Eph receptors exhibit context-dependent and sometimes opposing effects, playing dual roles as both promoters and inhibitors of cancer progression. The delicate equilibrium among signaling pathways, angiogenesis, and immune responses is crucial for maintaining tissue homeostasis. Dysregulation of this balance is implicated in various pathological conditions, especially cancer. The discrepancies observed in the roles of Eph receptors in cancer underscore the molecular mechanisms involved in ligand-dependent and ligand-independent signaling, as well as their influence on angiogenesis and immune response modulation. Despite substantial research, several key issues remain to be addressed. Clarifying the crosstalk between Eph receptors and other signaling pathways are essential for understanding the molecular mechanisms. Further research holds the potential to uncover novel therapeutic targets and strategies, especially when considering the diverse context in which Eph receptors operate. This exploration provides valuable insights, revolutionizing the approach to cancer therapy.

## Data Availability

No datasets were generated or analysed during the current study.
